# Maternal heavy alcohol use and toddler behavior problems: a fixed effects regression analysis

**DOI:** 10.1007/s00787-015-0677-5

**Published:** 2015-01-14

**Authors:** Ann Kristin Knudsen, Eivind Ystrom, Jens Christoffer Skogen, Leila Torgersen

**Affiliations:** 1 Department of Global Public Health and Primary Care, University of Bergen, Kalfarveien 31, 5018 Bergen, Norway; 2 Department of Health Registries, Norwegian Institute of Public Health, Bergen, Norway; 3 Department of Genetics, Environment and Mental Health, Norwegian Institute of Public Health, Oslo, Norway; 4 Department of Psychology, Faculty of Social Sciences, University of Oslo, Oslo, Norway; 5 Department of Public Mental Health, Norwegian Institute of Public Health, Bergen, Norway; 6 Drug Research Western Norway, Stavanger University Hospital, Stavanger, Norway; 7 Department of Psychosomatics and Health Behavior, Norwegian Institute of Public Health, Oslo, Norway

**Keywords:** Heavy alcohol use, Fixed effects models, Repeated measures, Behavior problems

## Abstract

Using data from the longitudinal Norwegian Mother and Child Cohort Study, the aims of the current study were to examine associations between postnatal maternal heavy alcohol use and toddler behavior problems, taking both observed and unobserved confounding factors into account by employing fixed effects regression models. Postnatal maternal heavy alcohol use (defined as drinking alcohol 4 or more times a week, or drinking 7 units or more per alcohol use episode) and toddler internalizing and externalizing behavior problems were assessed when the toddlers were aged 18 and 36 months. Maternal psychopathology, civil status and negative life events last year were included as time-variant covariates. Maternal heavy alcohol use was associated with toddler internalizing and externalizing behavior problems (*p* < 0.001) in the population when examined with generalized estimating equation models. The associations disappeared when observed and unobserved sources of confounding were taken into account in the fixed effects models [(*p* = 0.909 for externalizing behaviors (*b* = 0.002, SE = 0.021), *p* = 0.928 for internalizing behaviors (*b* = 0.002, SE = 0.023)], with an even further reduction of the estimates with the inclusion of time-variant confounders. No causal effect was found between postnatal maternal heavy alcohol use and toddler behavior problems. Increased levels of behavior problems among toddlers of heavy drinking mothers should therefore be attributed to other adverse characteristics associated with these mothers, toddlers and families. This should be taken into account when interventions aimed at at-risk families identified by maternal heavy alcohol use are planned and conducted.

## Background

One of the earliest developmental tasks is the evolvement of appropriate emotional and behavioral regulation in the toddler years [1]. The quality of the toddler’s maternal relationship and the social context under which the child grows up, are of extraordinary importance for optimal child development [2], but may be disturbed by maternal dysfunctional behavior [3]. One potentially important risk factor for maternal dysfunction in the toddler years is heavy alcohol use. Several studies have reported associations between heavy maternal alcohol use and increased levels of internalizing and externalizing behavior problems and lower social competence among school-age children [4–6], and adolescents and adults [7, 8]. Maternal risky alcohol use before [9] and during [10] pregnancy has also been linked to early development of behavior problems. However, in studies on toddlers and preschool children, the focus has primarily been on the impact of the father’s heavy alcohol use on toddler behavior problems [11–15]. Studies that have included the role of maternal heavy alcohol use have examined this in addition to heavy paternal alcohol use [11–13, 15], with equivocal findings in regard to whether maternal heavy alcohol use further increases the risk of adverse child outcomes. The independent role of maternal heavy alcohol use on behavior problems in the toddler years is thus largely unknown, and is therefore the focus of the present study.

Children who grow up in families where either one or both of the parents are heavy alcohol users are also exposed to a range of other factors that may have adverse impact on their function and development. Although associations between parental heavy alcohol use and child and adolescents behavioral problems have been reported in several studies, the extent to which these associations reflect causal linkages is not known. There are two different processes that could result in an observed association between maternal heavy alcohol use and toddler behavior problems. First, the association could reflect a cause and effect linkage. Alcohol affects emotion regulation, cognitive functioning, judgement and impulse control; factors that all are important when taking care of and raising children. By influencing these factors, heavy maternal alcohol use may have a direct impact on parenting behavior, for instance by reducing the ability of a timely, responsive and stable parenting of the child [16], which again may affect the child’s risk of developing behavior problems. Heavy maternal alcohol use may affect attachment quality [10] and reduce the mother’s awareness, attention and sensitivity towards her child [17]. Children of heavy drinking mothers may also experience inconsistent disciplining [17], and they are at increased risk of being exposed to neglect and abuse [18]. Thus, through the mediating effect of parenting behavior, heavy maternal alcohol use may have a causal effect on toddler behavior problems. One could also imagine situations where heavy maternal alcohol use has a direct impact on toddler behavior problems. Being exposed to an intoxicated mother may cause feelings of confusion, insecurity and fear in the child, for instance in situations where the intoxication has lead to child abuse or neglect [18]. In a sample of children whose fathers were in treatment for alcoholism, Andreas and O’Farrell [19] found that the level of child maladjustment varied according to the father’s current alcohol use status. The results from this study may indicate a causal role, in which parental heavy alcohol use acutely affect child behavior problems.

An alternative explanation is that the association between maternal heavy alcohol use and child behavior problems is non-causal, and rather reflects the presence of important confounders. A myriad of heritable [20], contextual [21], emotional [22] and behavioral factors associated with both heavy alcohol use and toddler behavior problems may be present in these mother–child constellations. Among the most acknowledged confounders in the association between maternal alcohol use and offspring outcomes are negative affectivity and maternal psychopathology [such as anxiety, depression and attention deficit hyperactivity disorder (ADHD)], outgoing personality, marital conflict, presence of a heavy drinking partner, and a generally more disorganized and chaotic lifestyle [11, 23–28]. Prenatal alcohol use is a particularly important factor associated with both postnatal alcohol use and child behavior problems [10]. Studies that have examined the effect of postnatal maternal alcohol use on offspring outcomes have been limited in their ability to control for this important confounder. Finally, alcohol use disorders includes a genetic component, and women engaging in risk drinking may also transfer genetic vulnerability to their children, which may be expressed as behavior problems in the child [20, 29]. It is extremely difficult to define and measure all these potential confounders exhaustively in a single epidemiological study [30]. As previous studies have had scarce access to data beyond exposure and outcome variables, they have not been able to separate a potential causal effect of maternal heavy alcohol use from confounding effects.

In the present study, we applied fixed effects models as a method that can mitigate the challenges of unmeasured confounders. In repeated measured data, fixed effects models can eliminate between-individual differences (such as genetic, contextual, functional or personality differences between mothers) and show the effect of within-individual changes (i.e., whether a change in heavy alcohol use in mother A is associated with changes in behavioral problems in toddler A). Sources of variations in exposures and outcomes present before and at the first measurement, and for which the effect is likely to be stable over time (“time-invariant factors”), are in this model considered as fixed at the first measurement, and can therefore be controlled for using the individual person as its own control [30]. Measuring of these potential confounders is therefore unnecessary. Fixed effects regression models thus estimate the within-individual exposure–outcome association, taking both observed and unobserved confounders that are specific to the individual participant into account [31]. However, heavy alcohol use is also associated with a disorganized and chaotic lifestyle, relationship instability and psychopathology [28]. Factors such as changes in maternal civil status, mental health, or recent experience of negative life events may also change between the measures, and affect both maternal heavy alcohol use status and toddler behavior problems. Such time-variant variables may also be controlled for in fixed effects regression models, and these models thus allows for the control of both time-invariant confounders and observed time-variant covariates.

By employing fixed effect regression models on a national representative sample of 51,000 children, followed from birth until age 36 months, we aimed in the present study to establish whether maternal heavy alcohol use and toddler behavioral problems are independently associated. This is an important step in ascertaining a potential causal linkage between maternal alcohol use and toddler behavior problem. More specifically, we wanted to examine: (1) whether an association between maternal heavy alcohol use and behavioral problems in the toddler years can be found in the general population, (2) whether an association between maternal heavy alcohol use and toddler behavioral problems are partly or fully explained by observed and unobserved sources of confounding, and (3) whether an association is further attenuated by taking the potential time-variant confounders of maternal mental distress, changes in civil status and experience of negative life events into account.

## Materials and methods

### Study design and participants

The data examined in the present study were derived from the Norwegian Mother and Child Cohort Study (MoBa). MoBa is a prospective population-based pregnancy cohort study conducted by the Norwegian Institute of Public Health [32]. Participants were recruited from all over Norway from 1999 to 2008, and women consented to participation in 40.6 % of the invited pregnancies. The cohort now includes 114,500 children, 95,200 mothers and 75,200 fathers.

The primary measures were conducted at gestation week 17/18, with follow-up by questionnaires at regular intervals. The current study is based on version seven of the quality-assured data files released for research in 2013. The current study has a repeated measures design, and employs information reported by the mothers when the child was 18 months (Questionnaire 5, Q5), and 36 months old (Questionnaire 6, Q6). The main inclusion criterion was valid information on the outcome measures in both questionnaires, fulfilled by *N* = 51,891 of the mothers. The second and third child in twin or triplet births (*n* = 770), and children with missing information on gender (*n* = 6) were excluded from the sample. The total population eligible for the current study was *N* = 51,115, 46.8 % of those who participated at least once in the MoBa study.

### Maternal heavy alcohol use

Mothers who either drank alcohol frequently, or who drank excessive quantities of alcohol when they were drinking, assessed at child age 18 and 36 months, were defined as heavy alcohol users. Frequency was measured with the item “How often do you consume alcohol?” with alternatives ranging from “never” to “roughly 6–7 times a week”, while quantity were assessed for both weekend and weekdays through the item “How many units do you usually drink when you consume alcohol?” with fixed answer categories ranging from “less than 1”, to “10 or more”. In MoBa, one unit was defined as equivalent to 1.5 cl (12.8 g) of pure alcohol.

Although not a fully valid measure of alcohol problems per se [33], frequency of alcohol use may be an indicator of alcohol abuse and dependence [34]. Among Norwegian women, alcohol use is most common in the weekend evenings [35]. Weekday alcohol use has been found to be more strongly associated with alcohol problems than weekend alcohol use [36], probably because those who engage in weekday alcohol use are more likely to be heavy drinkers [36]. We therefore categorized those who reported to be drinking alcohol four times a week or more as heavy drinkers in terms of frequency, as these mothers were likely to drink both during weekends and weekdays.

Risky single-occasion alcohol use among women is commonly referred to as the intake of approximately 40–60 g pure alcohol (≈3.1–4.7 in MoBa alcohol units) or more in a single occasion [34, 37]. However, the level of intoxication depend on factors such as duration of the drinking occasion, age, body weight and whether or not the alcohol is consumed together with food [37]. In the present context, we aimed to identify mothers truly at risk for intoxication, and we thus categorized mothers who reported that they usually drank seven or more alcohol units as “heavy drinkers”.

Heavy alcohol use in the present context was thus defined as (1) alcohol use four or more times a week, or (2) usually consumption of seven alcohol units or more when drinking. Approximately, 1 % of the mothers answered affirmatively on the first category, while 3 % of the mothers reported the second category.

### Toddler behavior problems

Toddler behavior problems were assessed in the 18- and 36-month questionnaires with items from the Child Behavior Checklist version for preschool children (CBCL/1.5–5) [38]. CBCL/1.5–5 is constructed to cover a range of emotional, social and behavior problems, and consists of 99 items describing child behavior in the preceding 2 months. CBCL/1.5–5 has several subscales, which again are included in two scales measuring internalizing and externalizing behavior problems. The CBCL version for older children has been validated in a Norwegian study [39], while the CBCL/1.5–5 has been validated in Dutch and Danish samples [40, 41].

In common with most large inter-disciplinary epidemiological studies, item selection was necessary due to space restriction in the MoBa questionnaires. The selected CBCL items represented subscales of the internalizing domain (“emotionally reactive,” “anxious/depressed,” and “somatic complaints”) and externalizing domain (“attention problems” and “aggressive behavior”). The item selection procedure was based on consensus among specialists in clinical and developmental psychology and aimed at representing each subscale with items that were both clinically and theoretically relevant. Thirteen of the items were included in both questionnaires, and were used in the current study as indicators of internalizing (five items) and externalizing behavior problems (eight items). The mothers were asked to rate each item on a scale from 0 “not true”, 1 “somewhat or sometimes true” to 2 “very true or often true”. Sum scores were calculated for each subscale. In cases were responses were missing for one or two items, sum scores were calculated based on the mean score on the items that were responded to. Scores for internalizing problems ranged from 0 to 10, while scores for externalizing problems range from 0 to 16. To assess changes in total scale scores that were not due to normative changes between 18 and 36 months, the scores in the internalizing and externalizing problem variables were standardized for each age group.

### Time-variant covariates

A selection of variables that appeared in the time-period between the 18- and 36-month assessments, and which were considered likely to affect both maternal heavy alcohol use and toddler behavior problems were included as potential time-variant confounders. These are presented below.

#### Maternal civil status

The mother was asked to report her current civil status. Based on her response, a dichotomous variable was conducted, indicating whether the mother was currently (0) in a relationship or (1) single.

#### Maternal mental distress 

Maternal mental distress was assessed with the eight items version of Hopkins Symptom Check-List (SCL-8) [42]. For each item, the respondents were asked to indicate how much any of the presented emotions and cognitions had bothered them during the past 2 weeks, scored on a four-point scale ranging from 1 “not bothered” to 4 “very bothered”. The item scores were then summarized into a continuous variable, ranging from 8 to 32. For participants with missing responses on one, two or three items, the sum score was based on the summarized mean value of the items responded to.

#### Negative life events

Four variables on negative life events last year were included (yes/no): (1) whether the mother herself had been seriously ill or injured, (2) whether anyone close to the mother had been seriously ill or injured, (3) whether the mother had been involved in a serious accident, fire or robbery, or (4) whether the mother had lost someone close to her.

### Statistical procedures

Missing responses in the exposure variables and time-variant variables ranged from 0.3 (civil status at 18 months) to 5.3 % (negative life events at 36 months). Multiple missing imputation procedures in Stata 12.0 [43], using the multivariate chained approach with ten imputation procedures, were employed before the main analyses were conducted.

The pooled population-averaged associations between maternal heavy alcohol use and age-standardized levels of internalizing and externalizing behaviors were investigated with generalized estimating equation (GEE) methods [44]. Next, we used fixed effects regression models to examine the within-individual associations between maternal heavy alcohol use and toddler behavior problems, controlled for observed and unobserved confounding factors. The use of a fixed effects model allows to estimate the effect of changes in maternal heavy alcohol use status from time 1 (18 months) to time 2 (36 months) on changes in toddlers level of behavioral problems, holding all the observed and unobserved sources of individual variation in heavy alcohol use status and toddler behavior problems at 18 months constant. Finally, we included the time-variant observed factors in the fully adjusted models. All associations were adjusted for pregnancy at the 18- or 36-month questionnaires. Estimates are presented as coefficients (*b*), with standard errors (SE). Statistical significance is reported with *p* values. All analyses were conducted in Stata 12.0 [45].

### Ethics

License to conduct the MoBa study was obtained from the Norwegian Data Inspectorate, and the current study was approved by the Regional Committee for Medical Research Ethics. Informed consent was obtained from all participants.

## Results

The characteristics of the sample before multiple missing imputation procedures, including some of the fixed characteristics at 18 months are presented in Table 1. When the toddler was aged 18 months, 4.3 % of the mothers reported heavy alcohol use, compared to 4.5 % at toddler age 36 months (Table 1). While 2 % of the samples were heavy drinkers at both assessments, slightly more than 2,300 mothers (4.7 %) changed heavy alcohol use status from 18 months to 36 months (data not shown). In the total sample, 2.2 % of the mothers ceased heavy alcohol use behavior while 2.5 % started using alcohol heavily from toddler age from 18 months to 36 months. Mean level of internalizing and externalizing behavior problems was similar at 18 and 36 months (Table 1). More mothers were single, had experienced a negative life event and had higher levels of mental distress when the toddler was 36 months compared to 18 months (Table 1).

**Table 1:** Descriptive characteristics on fixed effects at toddler age 18 months, and exposure variables, outcome variables and time-variant covariates of the MoBa participants at toddler age 18 and 36 months

	18 months		36 months	
	*n* (%)	Mean (SD)	*n* (%)	Mean (SD)
Fixed characteristics				
Child gender, boy^a^	25,006 (48.9)			
Birth weight (g)^a^		3,585.3 (562.9)		
Gestation length (weeks)^a^		39.5 (1.8)		
Maternal age at birth (years)^a^		30.4 (4.4)		
Number of older siblings^a^		0.7 (0.8)		
Completed education mother, high school or less^a^	14,584 (30.1)			
Household income before pregnancy, <400,000 NOK	10,416 (21.7)			
Alcohol consumption during pregnancy (yes)				
Week 0–12	13,306 (26.5)			
Week 13–24	4,444 (10.0)			
Week 25+	5,034 (10.0)			
Paternal heavy alcohol use^b,c^	8,710 (21.3)			
Exposure				
Maternal heavy alcohol use^b^	2,146 (4.3)		2,237 (4.5)	
Missing	694 (1.4)		1,445 (2.8)	
Outcomes				
Internalizing problems^d^		1.3 (1.2)		1.4 (1.4)
Externalizing problems^d^		3.9 (2.2)		3.9 (2.4)
Time-variant covariates				
Civil status (single)	1,645 (3.2)		2,214 (4.4)	
Missing	150 (0.3)		1,019 (2.0)	
Maternal mental distress^e^		10.2 (2.9)		10.2 (3.1)
Missing	569 (1.1)		1,040 (2.0)	
Negative life events last year (one or more)		12,672 (25.3)		12,859 (26.6)
Missing	1,098 (2.2)		2,727 (5.3)	

Characteristics only presented for participants with valid responses

Total sample size *n* = 51,115

^a^When the child is born

^b^Heavy alcohol use defined as (a) drinking alcohol four or more times a week, or (b) drinking seven or more alcohol units when they are drinking

^c^6 months before or during partner’s pregnancy

^d^Sum score, not age standardized

^e^Score on SCL-8

The results from the analyses employing GEE and fixed effects regression models are presented in Table 2. The GEE models indicated a positive association in the population between maternal heavy alcohol use and toddler behavior problems (*p* < 0.001 for both internalizing (*b* = 0.171, SE = 0.015) and externalizing behaviors (*b* = 0.111, SE = 0.015). However, the associations disappeared when observed and non-observed sources of confounding were taken into account in the fixed effects models [(*p* = 0.909 for externalizing behaviors (*b* = 0.002, SE = 0.021), *p* = 0.928 for internalizing behaviors (*b* = 0.002, SE = 0.023)], with an even further reduction of the estimates when maternal civil status, maternal mental distress and experience of negative life events were included in the model.

**Table 2:** Associations between maternal heavy alcohol use and toddler externalizing and internalizing behaviors

	Externalizing behaviors^c^		Internalizing behaviors^c^	
	*b* (SE)	*p*	*b* (SE)	*P*
Pregnancy adjusted^a^	0.171 (0.015)	<0.001	0.111 (0.015)	<0.001
Fixed effects model	0.002 (0.021)	0.909	0.002 (0.023)	0.928
Adjusted for fixed effects and time-variant covariates^b^	−0.001 (0.021)	0.968	−0.003 (0.023)	0.894

Regression analyses adjusted for pregnancy, fixed effects, and fixed effects and time-variant covariates

^a^Generalized estimating equation (GEE) model

^b^Adjusted for concurrent maternal civil status, concurrent maternal mental distress and concurrent maternal experience of negative life events last year

^c^Age standardized

## Discussion

Based on a large birth cohort from the general population, we found that the average toddler of a heavy alcohol use mother had heightened levels of behavior problems. On the individual level, however, we found no indication of a causal effect between maternal heavy alcohol use and toddler behavior problems, as the increased levels of toddler behavior problems found in the population were completely explained by controlling for observed and unobserved confounders in a fixed effect model.

Although the need for knowledge about the effect of postnatal maternal heavy alcohol use on child outcomes has been emphasized [10], the literature in this area is scarce. Research that has examined the effect of parental heavy alcohol use on behavior problems in toddlers is in general limited, and the role of heavy alcohol use toddler mothers has, to the best of our knowledge, not been studied independently of paternal heavy alcohol use. Previous studies have indicated that toddlers and children whose both parents or fathers engage in heavy alcohol use have more behavior problems [4, 5, 11–15]. In the present study, we found higher levels of externalizing and internalizing behavior problems in the population among toddlers of mothers who engaged in heavy alcohol use. The increased problems were, however, completely explained by observed and unobserved sources of confounding. This finding is in line with studies examining the genetic versus the environmental risk for internalizing and externalizing behavior problems in adolescent children of alcohol abusing parents, using the children-of-twins (COT) design. A recent systematic review of these studies by McAdams and colleagues concluded that parental alcohol abuse had no significant environmental influence on offspring mental problems in adolescence [46]. Although toddlers in our sample of heavy alcohol use mothers are at increased risk, this risk seems to be related to other adverse characteristics of the mother, family or toddler and not to the heavy alcohol use per se.

Our findings indicates not only lack of effect of maternal heavy alcohol use on toddler behavior problems, but also a lack of effect from other factors that may temporarily or acutely be affected by heavy maternal alcohol use, such as parenting behavior, attention, patience, responsiveness or warmth toward the child. However, as fixed effects models are developed to examine the effect of within-individual changes (i.e., the effect of changes from low/moderate to heavy alcohol use), we cannot study the effect of stable heavy drinking in these models. Further, we did not have information on to what degree the toddlers had been exposed to the mothers heavy drinking. The maternal heavy alcohol use may have only taken place when the toddler was not present, for instance after bedtime or when the mother had a babysitter. A higher level of family functioning and positive parenting styles may also have been protective for the negative effect of heavy maternal alcohol use [47]. We also did not have information about the duration of the period in which the mother engaged in heavy alcohol use. We can thus not exclude the possibility that toddlers who are directly and over time exposed to a heavy drinking mother will show different behavior patterns than found in our study.

The increased level of behavior problems identified at the population level among toddlers of heavy alcohol use mothers could have practical implications. Maternal heavy alcohol use is a potential indicator for vulnerable families and toddlers at risk, even if the increased risk is completely explained by other adverse factors. Screening for alcohol problems is part of routine assessments for pregnant women, and could easily be included as part of the general postnatal assessment. Interventions for families with heavy alcohol use mothers should take a broad perspective, and be aimed at a range of potential adverse characteristic. Based on our results, it is unlikely that interventions aimed to only help the mother to cut-down her alcohol use will have an effect on the toddler’s behavior problems.

### Strengths and limitations

There are three important sources of bias in epidemiological research: confounding, measurement errors and selective participation. The main strength of the present study is the ability to control for all potential confounders, both observed and unobserved, at the 18-month assessment with the use of a repeated measures design analyzed with fixed effects models. This method made it possible for ascertaining a potential causal linkage between maternal alcohol use and toddler behavior problem. Another strength is the large sample size taken from a general population of toddler mothers, which allowed us to study the effect of heavy maternal alcohol use in the general population, and not only among clinical cases.

Despite that the fixed effects model allows for control of all confounding factors up to age 18 months that has effect on both time points, some factors may still operate as time-specific confounders at 18 and 36 months. We controlled for three such sources of confounders; namely changes in maternal civil status, changes in maternal mental distress and experience of negative life events between the 18- and 36-months assessments. There may also be other factors operating as residual confounders. One such factor is changes in paternal alcohol consumption, which may be associated with both heavy maternal drinking and behavior problems in the offspring [10, 12, 13, 25, 48]. Unfortunately, we did not have information on paternal alcohol consumption at toddler age 18 and 36 months, but the stable effect of paternal drinking was adjusted for in the fixed effects analyses. However, as no association was left after controlling for the fixed effects at 18 months, including changes in paternal alcohol consumption would probably not have any important impact on the estimates found.

With regard to the other two sources of bias, the present study has some limitations. Under-reporting of alcohol consumption is a challenge in health studies [49] and is particularly common in studies based on pregnancy samples [50]. Thus, some individuals may have been misclassified as non-heavy drinkers in the present study. Our strict definition of heavy alcohol use in the present study (four or more times a week, or more than seven alcohol units when drinking) was constructed to identify mothers reporting the highest levels of alcohol consumption. Despite this strict definition, no effect was found on our outcome measures. As only 4 % of the participating mothers satisfied our criteria, an increased threshold of heavy alcohol use may be of little public health relevance.

Even when employing a repeated measurement design with a time interval of 18 months, some cases of maternal heavy alcohol use may only have been present in the interval between the measurement points, and therefore not identified at the time of measurements. There is, however, no reason to believe that these mothers differ systematically from the mothers with heavy alcohol use at the measurement time points.

Another source of measurement error may be the non-inclusion of the full CBCL 1.5/5 in the MoBa questionnaires. While the items indicating behavior problems have been used previous publications from the MoBa database [9, 51], the abbreviated versions of the original scales are not validated. We aimed to reduce this potential measurement bias by constructing age-standardized continuous scales of internalizing and externalizing behavior problems, and examine a dose–response relationship without need for a validated cutoff.

Selective participation is the third important source of bias in epidemiological studies. The results in the present study are based on data from an extensive longitudinal study with several repeated measures. Continued participation may be associated with a high level of general functioning, and heavy alcohol use mothers who participated in MoBa may therefore differ from nonparticipating heavy alcohol use mothers [52]. However, a study of self-selection in MoBa concluded that the survey was relatively representative for the total population of pregnant women in Norway [53], and that selective nonparticipation was more likely to affect prevalence estimates than exposure–outcome associations. Further, the use of fixed effects models, with its focus on individual changes and control for all background variables, reduces bias associated with selective participation. Selection bias may therefore be of less of concern in the present study.

Finally, some limitations are related to the use of fixed effects models. Fixed effects models take only information on within-individual differences into account, discarding all information on between-individuals differences. If predictor variables vary greatly across, but not within, individuals over time, the fixed effect estimates become quite imprecise. Fixed effects models often produce estimates with large standard errors, leading to higher *p* values and wider confidence intervals [54]. About 2,300 (4.7 %) mothers changed their heavy alcohol use behavior from child age 18 to 36 months in the present study, and this should be a large enough sample to detect any effect of changes in alcohol use on toddler behavior problems. With standard errors in the fully adjusted fixed effects models at 0.021 for externalizing and 0.023 for internalizing behaviors, the true causal effects of maternal heavy alcohol use would have to be less than approximately 0.04 to not reach a *p* value of <0.05. This would represent effects of little clinical or public health importance.

## Conclusion

Increased levels of behavior problems among toddlers of mothers engaging in heavy alcohol use are probably not caused by the heavy alcohol use behavior per se, but should rather be attributed to a range of challenges and problematic characteristics associated with these mothers, toddlers and families. This should be taken into account when interventions aimed at at-risk families identified by maternal heavy alcohol use are planned and conducted.
